# A revalidation and critique of assumptions about urinary sample collection methods, specimen quality and contamination

**DOI:** 10.1007/s00192-020-04272-x

**Published:** 2020-03-05

**Authors:** Linda Collins, Sanchutha Sathiananthamoorthy, Jennifer Rohn, James Malone-Lee

**Affiliations:** 1 School of Nursing, Kingston University, Frank Lampl Building, Kingston Hill Campus, London, UK; 2 Department of Renal Medicine, Division of Medicine, University College London, London, UK

**Keywords:** Midstream, Urine, Sample, Catheter, Peezy, UTI

## Abstract

**Introduction and hypothesis:**

Midstream urine (MSU) is key in assessing lower urinary tract syndrome (LUTS), but contingent on some assumptions. The aim of this study was to compare the occurrence of contamination and the quality of substrates obtained from four different collections: MSU, catheter specimen urine (CSU), a commercial MSU collecting device (Peezy) and a natural void. Contamination was quantified by differential, uroplakin-positive, urothelial cell counts.

**Methods:**

This was a single blind, crossover study conducted in two phases. First, we compared the MSU with CSU using urine culture, pyuria counts and differential counting of epithelial cells after immunofluorescence staining for uroplakin III (UP3). Second, we compared the three non-invasive (MSU, Peezy MSU™, natural void) methods using UP3 antibody staining only.

**Results:**

The natural void was best at collecting bladder urinary sediment, with the majority of epithelial cells present derived from the urinary tract. CSU sampling missed much of the urinary sediment and showed sparse culture results. Finally, the MSU collection methods did not capture much of the bladder sediment.

**Conclusion:**

We found little evidence for contamination with the four methods. Natural void was the best method for harvesting shed urothelial cells and white blood cells. It provides a richer sample of the inflammatory exudate, including parasitised urothelial cells and the microbial substrate. However, if the midstream sample is believed to be important, the MSU collection device is advantageous.

## Introduction

A urinary tract infection (UTI) is the leading reason why patients seek treatment in primary care [1]. One in three women will be treated with antibiotics for UTI by the age of 24 and 40% to 50% of women will experience one or more UTIs in their lifetime, with 10–15% experiencing recurrent infections [2]. A UTI is commonly identified among young adolescents attending sexual health centres and accounts for 17% of treatment cases [3]. In the adult population of 65 years and older, a UTI is the second most common cause of infectious disease related to hospitalisations in the USA [4]. A UTI is a debilitating condition causing the onset of painful urination (dysuria), increased urinary frequency, the inability to start urinating (hesitancy) and the sensation of a sudden need to urinate (urgency) [5], all of which are classified as lower urinary tract symptoms (LUTS) [6].

Many clinical guidelines advocate urinary dipstick testing for leucocyte esterase and nitrite as a means of detecting UTI, but evidence suggests that their utility might be limited [7]. Dipstick analyses are surrogate tests, referenced not to microscopic pyuria but to a gold standard urine culture threshold for UTI, which guidelines accept as being between 10^3^ cfu ml^−1^ and 10^6^ cfu ml^−1^ of the pure growth of a single urinary pathogen [8]. This test has attracted criticism too [9–11]. Furthermore, the literature shows that the methods of sample collection for urine culture have never been validated in appropriate clinical trials. Justification for the use of such methods is based on plausible assumptions without supporting evidence. It is now recognised that routine urinalyses, including dipstick and culture, are insensitive, thus missing genuine infection in many symptomatic patients [12–14].

There are three commonly used urine collection methods: the midstream clean catch technique (MSU) [15], the catheter specimen of urine (CSU) [16], and suprapubic aspiration [17]. Sample contamination is a key concern, particularly with regard to MSU. Our understanding is hampered by the variable definitions of “contamination” described in the literature. Some have claimed that contamination is indicated by isolation of microbes typical of skin flora, such as *Corynebacterium* and *Staphylococcus* [18–20]. According to Collier et al. [21], contaminated urine samples are revealed by finding squamous epithelial cells on urine microscopy. Wilson and Gaido [22] defined contamination as ≥2 different types of organisms at >10^5^ ml^−1^ or 1 organism at <10^4^ ml^−1^. Others have reported urine samples as contaminated, without describing the criteria used [23, 24]. These disparities add to the confusion that affects these diagnostic methods and there are no data available to aid clarification. An important consideration is the substrate that these collection methods should seek to obtain for culture. Historically, the emphasis has been on uncontaminated urine samples being cell-free [22]. However, contemporary studies have shown that UTI is associated with microbial parasitisation of urothelial cells, via surface attachment and/or intracellular invasion [25–28]. This parasitisation stimulates urothelial cell shedding as part of an innate immune response [27]. It has been found that the urinary urothelial cell counts are elevated in association with other markers of infection and that the proportion of parasitised cells among the cell sediment increases during UTIs, along with inflammatory markers and changes in the urinary microbiome [13, 27, 29]. If the primary pathology is the microbial invasion of urothelial cells, stimulating the concomitant innate immune response of increased urothelial cell shedding, it is plausible that these cells would make a better substrate for urine culture than would supernatants of planktonic bacteria. This was demonstrated in our previous work [12].

Shed urothelial cells are an attractive option for study when seeking to examine contamination with different sampling methods. Uroplakin III (UP3), a transmembrane protein found exclusively in the urinary tract [30], is a useful biomarker for discerning the origin of epithelial cells in a urine sample. Thus, it is possible to discriminate contaminating cutaneous and vaginal squamous cells from urothelial umbrella cells using a specific antibody against this protein with immunofluorescence staining protocols.

This study consisted of two parts. The first was a comparative study of the urinalysis results obtained from samples collected by MSU and CSU. It was a random allocation, cross-over design and the urine sampling performances were compared using microscopic pyuria counts, epithelial cell counts, microbial growth from spun cell sediment culture and UP3-positive cell counts to measure contamination. The second part of the study was a random allocation, cross-over comparison study of contamination obtained with MSU, a novel MSU sampling device called a “Peezy” and naturally voided urine, in which we measured microscopic epithelial cell counts using UP3 staining.

## Materials and methods

This study was presented to the National Research Ethics Service Committee (NRES) in Harrow, London, UK, to obtain approval to conduct the investigation. Ethical approval was granted, which signified that this study complied with conditions that were favourable and worthy of safe research practice. Ethical approval was given subject to all clinicians in the study having undergone training in good clinical practice (GCP). The two-part comparative studies began in January 2013 and lasted until March 2015. Patients with LUTS attending the Community LUTS Clinic, Hornsey Central Health Centre, London, UK, made up the participant population. The patient sample was drawn from women diagnosed with chronic UTI, painful bladder syndrome (PBS), overactive bladder (OAB) and general LUTS.

Patients were given an information sheet about the study and were offered the opportunity to ask questions and address any concerns. The first part of the study was designed to ascertain whether there were outcome signals that merited a focused analysis of urinary epithelial cells in relation to sample collection. On the day of recruitment, patients were invited to the centre, and written informed consent was obtained. Each participant was given a unique non-identifiable number. All patients completed a validated female LUTS questionnaire (FLUTS and FLUTSqol) [31] and provided MSU and CSU samples. The MSU method was accomplished by the patient spreading her labia apart with one hand and then wiping the urethra area with moistened wipes using the other hand [20]; the CSU was achieved by placing the patient in the lithotomy position, inserting the urethral catheter along the urethra and into the bladder to collect part of the urine outflow [32].

**Fig. 1:**
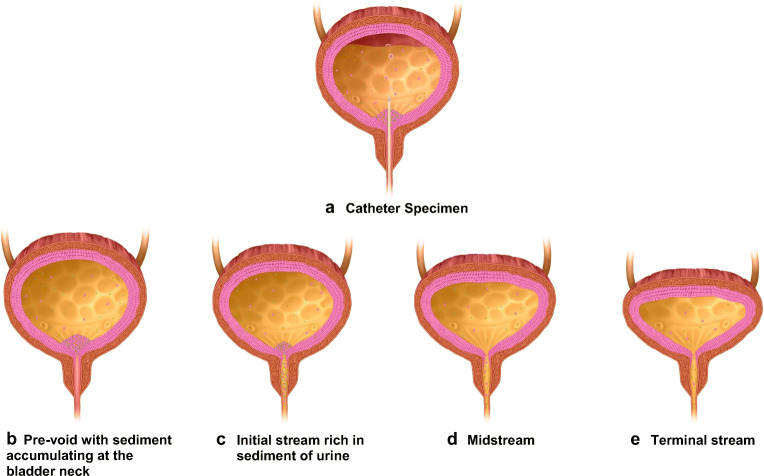
**a** Illustration of the fact that the cellular sediment collects at the bladder base under the influence of gravity. A catheter passes through this sediment to obtain a specimen from the urine that is above this substrate. The sequence in **b–e** illustrates the phases of the voiding processes. The inflammatory sediment is shown accumulating at the bladder neck under the influence of gravity. The first part of the stream contains the largest quantity from the sediment at the bladder neck and this reduces over the course of the voiding process

The substrate of interest in urinalysis is the inflammatory exudate, which contains microbes, white cells, shed urothelial cells and other debris. This may become contaminated with external debris during collection. The collection methods sample different parts of the bladder urine (Fig. 1). The presence of sediment and contaminants may be influenced by the different collection methods. Thus, we should seek to examine the markers that reflect these different elements.

The urine sampling methods were randomly sequenced and obtained with a 1-h interval between specimen collections. The specimen volume requirement was small and well within the capacity of an hour. Urine aliquots were obtained from the unspun specimens and these were introduced, unstained, into a Neubauer counting chamber; a microscope was then used to count the white blood cells (WBCs), red blood cells (RBCs) and epithelial cells. A sediment culture [12] was carried out. All urine samples were collected in a 30-ml container and spun down in a Denley refrigerated centrifuge at 800 rpm (~75 relative centrifugal force [RCF]). The remaining cell pellet was re-suspended in phosphate-buffered saline (PBS) and three ten-fold serial dilutions were made in PBS. A volume of 50 μl of the re-suspended pellet and dilutions was plated onto chromogenic CPS3 (now renamed CPS-Elite) agar (bioMérieux, France) [33] and dispersed evenly using a spreader. The culture plates were incubated aerobically at 37 °C for 24 h, followed by colony enumeration and identification using the Chrom ID™ colour chart provided.

An aliquot of spun sediment cells was collected and stained for UP3 as described previously [27]. Briefly, epithelial cells were adhered to a glass slide using a Shandon Cytospin Cytocentrifuge (Thermo Scientific) with 80 μl of a urine sample for 5 min at 75 RCF. The cells were fixed with 4% formaldehyde in PBS for 15 min, blocked with 10% normal goat serum for 30 min and stained for 1 h with anti-UP3 antibody. After three washes with PBS, the secondary antibody (goat anti-mouse IgG conjugated to Alexa-flour 488) was applied for 40 min, washed three times and the DNA stain 4′,6-diamidino-2-phenylindole (DAPI) was later applied for 20 min before mounting.

The second part of the study was conducted in the wake of the first experiment and was designed to compare the quantitative and qualitative properties of epithelial cells captured in specimens obtained from an MSU, an MSU collection device (Peezy) and a naturally voided specimen, which was a straightforward urine sample collected by the patient in a container without a technique. The Peezy MSU™ is a urine collection method achieved by urinating into an engineered device [21]. The patients were instructed to wash their hands, clean the genital area with wet wipes, attach the collection bottle to the Peezy and position the Peezy MSU™ device against the perineum whilst passing urine. As the patient began to pass urine, the first part of the stream entered the funnel and caused a sponge valve to swell and block flow through the main funnel exit. Once the blockage was established, a midstream specimen accumulated in the funnel and was passed out of an overflow side drain into the universal container. The Peezy MSU™ device was then discarded into the clinical waste bin. All urine samples were obtained using each method, through random sequencing, with one method each day over 3 consecutive days.

This second study commenced in August 2014. Patients recruited from the first study were asked to participate in the second part, but fresh consent was obtained. A random code dictated the order of sampling, which occurred on the first day of the 3 consecutive days. For experiment 2, samples were used to examine the epithelial cell content using the same methods as in experiment 1.

### Sample size

The sample was calculated using the G Power software package. The effect size was set as Cohen’s d = 0.55; α = 0.05; power (1 – β err probability) = 0.8; non-centrality parameter δ = 2.9; critical t = 2; df = 27; sample size = 28; actual power = 0.8 or 80%.

### Statistical analysis

The data were not normally distributed and exhibited wide variance; thus, to analyse quantitative measures we used the non-parametric Friedman test, which achieves a one-way analysis of variance by ranks. This is an alternative to the Kruskal–Wallis when analysing repeated measures. We used the Chi-squared test to analyse the differences in proportions.

## Results

### Demography and symptoms

In the first study, 60 female adult patients were enrolled. The mean age of the patient group was 60 years (SD = 12). The patients suffered from chronic LUTS and were being treated for chronic UTI. The demographic data are shown in Table 1 and demonstrate that the patients were suffering from significant symptoms.

**Table 1:** The average symptom scores and quality of life scores for patients who attended the Community Lower Urinary Tract Symptom (LUTS) Clinic with LUTS

Participants	Mean age	Mean urgency [34] score (maximum = 64) Normal = 0 [35]	Mean pain score [34] (maximum = 12) Normal = 0 [35]	FLUTS [34] (without urgency and pain) (maximum = 149) Normal = 0 [35]	FLUTSqol [36] (maximum = 288) Normal = N/A
Patients	60 (SD = 12)	16 (SD = 11)	10 (SD = 7)	64 (SD = 40)	151 (SD = 75)

### Evaluation of pyuria, epithelial cells and UP3-positive cells

To evaluate the presence of pyuria, epithelial cells and UP3-positive cells, a total of 118 urine samples were examined (MSU = 60, CSU = 58). The patients were at various stages of disease. The data analysis results are shown in Tables 2, 3, 4, and 5. Microscopic pyuria counts (log_10_ WBC μl^−1^) were significantly lower in CSU compared with MSU. Similarly, there were greater numbers of epithelial cells in MSU, the majority of which were UP3-positive, indicating urothelial origin. There was no significant difference in the proportion of UP3-positive cells between the MSU (0.81) and CSU (0.9) methods. If the CSU method collected a significantly less contaminated sample, the proportion would be expected to be significantly higher with the CSU method. These results suggest that MSU might enrich a urine sample for urinary tract cell sediment compared with the CSU, without increased or appreciable contamination.

**Table 2:** Evaluation of microscopic pyuria for the midstream urine (MSU) and catheter specimen urine (CSU) samples

Analysis	Pyuria count MSU (WBCs μl^−1^)	Pyuria count CSU (WBCs μl^−1^)	*p*
Mean	65	24	0.001
Median	0	0	
Standard deviation	321	148	

*WBCs* white blood cells

**Table 3:** Evaluation of microscopic urinary epithelial cells for the midstream urine (MSU) and catheter specimen urine (CSU) samples

Analysis	Epithelial cells (μl^−1^) MSU	Epithelial cells CSU (μl^−1^)	*p*
Mean	21	2	0.002
Median	2	0	
Standard deviation	103	4

**Table 4:** Evaluation of uroplakin-positive cells (UP3) for the midstream urine (MSU) and catheter specimen urine (CSU) samples

Analysis	UP3 cells (cells/80 μl) MSU	UP3 cells (cells/80 μl) CSU	*p*
Mean	17	5	0.006
Median	3	1	
Standard deviation	30	12

**Table 5:** Evaluation of the proportion of uroplakin-positive cells (UP3) for the MSU and CSU sample

Analysis	Proportion of cells UP3-positive MSU	Proportion of cells UP3-positive CSU	*p*
Mean	0.81	0.9	0.98
Median	1	1	
Standard deviation	0.3	0.2	

### Evaluation of culture results

All 118 urine samples were sent to the National Health Service (NHS) laboratory for routine culture (plating of 1 μl of urinary supernatant) and of these, 110 specimens were reported as “negative” (defined as below the 10^5^cfu/ml threshold) or “mixed growth”, which is commonly reported for chronic UTI patients [12, 13, 37]. Thus, in addition to routine culture, we enriched infected cells by performing a spun sediment culture [12] on the samples. Table 6 describes the occurrences of the top 90% of isolates across all patients cultured from the three different specimen collection methods. The data show that the spun sediment culture performed on MSU samples was the most productive method and the technique performed with CSU samples had significantly more negative results. The standard NHS culture method was far less productive whether it was from MSU or CSU; taken together, these results emphasise the insensitivities of both collection methods. The CSU seems to trade purity for sensitivity.

**Table 6:** Results of the spun urinary sediment culture carried out at the community LUTS clinic laboratory and NHS laboratory

	Number (%) culturing *E coli*	Number culturing *Enterococcus*	Number culturing *Streptococcus*	Number culturing *Staphylococcus*	Number culturing *Proteus*	Number culturing yeast	Number with no growth	Total
MSU spun sediment culture, LUTS laboratory	18 (30)	13 (22)	12 (20)	5 (8)	8 (13)	4 (7)	0 (0)	60
CSU spun sediment culture, LUTS laboratory	4 (7)	15 (26)	1 (2)	0	2 (3)	5 (9)	31 (53)	58
NHS MSU laboratory results	1 (1)	2 (2)	1 (1)	0	0	1 (1)	100 (95)	105
NHS CSU laboratory results	1 (8)	2 (15)	0	0	0	0	10 (77)	13

### Evaluation of UP3-positive cells, comparing MSU, Peezy MSU™ and natural void

Thirty-one patients were enrolled in this study, which used the UP3-positive properties of urothelial cells to identify the origin of epithelial cells, given that skin and vaginal cells are UP3-negative. The mean age of the patients was 62 (SD = 10). In total, 93 non-invasive urine samples were collected by MSU, Peezy MSU™ and natural voided urine. Tables 7, 8, and 9 reports the analysis of the UP3-positive cells. The mean count of UP3-positive cells from the natural void method was greatest compared with the MSU and Peezy MSU™. Thus, natural void achieved the greatest abundance of the target substrate. The lowest UP3-positive cell count was observed with the Peezy technique; thus, this method captured the substrate the least. The UP3-negative cells were sparse, with no difference between specimen collection techniques (Table 7, 8, and 9). Thus, the proportion of all cells that were UP3-positive did not differ between the sampling methods, which implies that, contrary to assumptions, contamination by extra-urinary tract cells is not influenced by the sampling technique and was low overall.

**Table 7:** Evaluation of UP3-positive cells from midstream urine (MSU), Peezy MSU™ and natural void, which are all non-invasive urine collection methods

	UP3-positive cells MSU (cells/80 μl)	UP3-positive cells Peezy MSU™ (cells/80 μl)	UP3-positive cells Natural void (cells/80 μl)	*p*		
Analysis						
Mean	8	3	16	0.001		
Median	2	2	3			
Standard deviation	17	8	27			
Test statistics						
*N*					31	
Chi-squared					14.041	
df					2

**Table 8:** Evaluation of UP3-negative cells from midstream urine (MSU), Peezy MSU™ and natural void, which are all non-invasive urine collection methods

	UP3-negative cells MSU (cells/80 μl)	UP3-negative cells Peezy MSU™ (cells/80 μl)	UP3-negative cells natural void (cells/80 μl)	*p*	
Analysis					
Mean	1	0.87	3	0.325	
Median	0	0	0		
Standard deviation	2	2	9		
Test statistics					
*N*					31
Chi-squared					2.250
df					2

**Table 9:** Evaluation of the proportion of UP3-positive cells from midstream urine (MSU), Peezy MSU™ and natural void, which are all non-invasive urine collection methods

	Proportion of UP3-positive cells MSU (cells/80 μl)	Proportion UP3-positive cells Peezy MSU™ (cells/80 μl)	Proportion UP3-positive cells natural void (cells/80 μl)	*p*	
Analysis					
Mean	0.91	0.88	0.91	0.5	
Median	1.0	1.0	0.97		
Standard deviation	0.14	0.16	0.11		
Test statistics					
*N*					31
Chi-squared					1.5
df					2

## Discussion

The purpose of this study was to measure contamination and substrate content for four different methods of obtaining urine samples. It also used the fact that urothelial cells can be distinguished from squamous epithelial cells by UP3 staining. This enabled measurement of contamination by the extra-urinary milieu compromising these various methods. The proportion of UP3-positive cells found in MSU samples was previously reported as 75% (Q1 = 68, Q3 = 78.5) by Horsley et al [27]. This study had a similar outcome, and has extended this to three other sample collection methods. Taken together with a wide body of literature demonstrating that urothelial cell shedding is a common innate immune response to infection, this study suggests that the widely held assumption that epithelial cells in a urine sample indicate contamination might need to be revised [26]. The sample collection method did influence the absolute epithelial cell counts, the lowest number with CSU samples (Fig. 1a) and the greatest number with natural void (Fig. 1c), during which the bladder contracts and collapses to eliminate bladder urinary substrate. The Peezy device seemed to do what it claimed, achieving a sample of fewer cells and very few uroplakin-negative cells. However, the sediment trapped in the sponge valve of the Peezy device may, counter-intuitively, prove to be a richer substrate for microbiological investigation, as bacteria are known to adhere to and colonise the urothelial cells.

The rationale of midstream sampling has always been the avoidance of contamination. This was never properly tested and relied on assumptions. In this experiment we were able to analyse contamination directly by using UP3 staining of the epithelial cells as a proxy, and we found that contrary to what has been assumed, the majority of epithelial cells in all samples originated from the urinary tract.

The standard NHS MSU culture achieved a very small number of positive cultures, in contrast to the spun sediment cultures, despite the patients exhibiting significantly fewer urinary tract symptoms; this discrepancy has been reported previously by our team and others [38]. Urinary microscopic detection of pyuria and increased urothelial cell shedding provided strong evidence for the presence of UTI. The sediment culture generated a greater abundance of isolates. However, given that healthy urine is known to harbour many species of bacteria, including known uropathogens [39], it is not known whether these microbes are pathogenic or harmless commensals, a problem affecting all current urinary microbial detection methods.

The symptomatic measurements pain, urgency, FLUTS and LUTSqol were concordant with the pyuria and culture data [13]. It should be appreciated that CSU sampling missed a pathological signal in a significant proportion of patients with appropriate symptoms, pyuria and urothelial cell shedding. Compared with CSU, the MSU technique provided a more substantial sample of the inflammatory sediment. UTI involves colonisation of the urothelial cells by pathogenic microbes [12, 27, 40]. The innate immune response to this microbial colonisation is increased urothelial cell shedding, which may be promoted by mast cells [41]. The shed cells may be colonised or unaffected, but the proportion of parasitised cells in the face of infection would be expected to increase [42]. This sediment, a mixture of white cells, epithelial cells and debris, is likely to collect under the influence of gravity at the bladder base, forming a sampling target that should not be influenced by dilution effects. This is made evident by the characteristic milky quality of the terminal flow when a catheter is used to drain an infected bladder. A catheter, as well as a suprapubic stab, samples urine above this collection at the bladder base (Fig. 1a), and therefore contains fewer epithelial cells, white cells and microbes, a point reflected in the data reported here. Thus, a CSU sample may be an inferior option because it may miss a substantial amount of the pathology.

With the advent of more sensitive genomic technology, a number of groups have reported that the healthy bladder is not sterile, and that polymicrobial colonisation is the norm in both healthy and infected bladders [12, 14, 43]. Polymicrobial growth in urine specimens has been associated with LUTS in a recent study of similar patients [21]. Indeed, the sediment cultures carried out on urine samples collected by MSU and CSU in this study also demonstrated polymicrobial growth. In patients with symptoms of UTI, the species dispersion is much wider than in asymptomatic controls [12, 18, 43–45]. At this time it is not known what species of these mixes are responsible for the disease. In this study, the data on the UP3-positive cells argued against the common assumption that polymicrobial results and/or epithelial cells are pointers to contamination. A recent study has shown the isolation of a different microbial population using the Peezy compared with one isolated from peri-urethral swabs, based on the assumption of peri-urethral contamination of MSU samples [46].

The natural void provided the greatest number of urothelial cells and even in this situation, UP3 analysis did not show evidence of increased contamination, which contradicts long-held assumptions about urine sampling. The natural void captures the first part of the urinary stream; the influence of gravity would encourage such samples to contain a larger proportion of the sediment because of settlement at the bladder base (Fig. 1b). By avoiding the initial stream, the MSU and Peezy MSU™ methods collected samples containing fewer cells, the Peezy MSU™ providing the clearest specimens (Fig. 1d). The remaining urine in the bladder proceeds with a terminal flow, with very few bladder sediments (Fig. 1e).

The Peezy MSU™ device works with a sponge in the funnel exit, which inflates gradually on contact with the first part of the urinary stream, eventually blocking the outflow so that the remainder of the stream escapes by a side channel into a collecting tube. Thus, the bulk of the sediment deposit from the bladder neck would be expected to be trapped in the sponge. If the bladder base sediment is indeed, as recent work suggests, the best specific target for diagnostic analysis, so is the content of the sponge. As matters stand, the Peezy MSU™ achieves what it sets out to do and provides an MSU sample free of early stream content, but in achieving that goal, it is less effective at capturing the sediment at the base of the bladder.

In conclusion, this study, supported by previous findings [27], suggests that epithelial cells might be a legitimate component of the urinary sample and are not derived from the peri-urethral area. Naturally voided urine provides the richest source of urinary sediment and may be the better sampling method for urinalysis in the face of LUTS. By comparison, the MSU, and particularly the CSU, provide inferior samples, because the pathological cellular exudate is excluded.
